# Effectiveness of seasonal malaria chemoprevention in three regions of Togo: a population-based longitudinal study from 2013 to 2020

**DOI:** 10.1186/s12936-022-04434-w

**Published:** 2022-12-31

**Authors:** Tchaa A. Bakai, Anne Thomas, Jean Iwaz, Tinah Atcha-Oubou, Tchassama Tchadjobo, Nagham Khanafer, Muriel Rabilloud, Nicolas Voirin

**Affiliations:** 1 Université de Lyon, Lyon, France; 2 Université Lyon 1, 69100 Villeurbanne, France; 3 Service de Biostatistique-Bioinformatique, Pôle Santé Publique, Hospices Civils de Lyon, 69003 Lyon, France; 4 Équipe Biostatistique-Santé, Laboratoire de Biométrie et Biologie Évolutive, CNRS UMR 5558, 69100 Villeurbanne, France; 5 EPIMOD (Epidemiology and Modelling in Infectious Diseases), 01240 Lent, France; 6 Programme National de Lutte contre le Paludisme (PNLP), 01 BP 518, Lomé, Togo; 7 Service d’Hygiène, Épidémiologie et Prévention, Hôpital Édouard Herriot, Hospices Civils de Lyon, 69003 Lyon, France

**Keywords:** Seasonal malaria chemoprevention, Effectiveness, Children, Togo

## Abstract

**Background:**

In 2012, the World Health Organization (WHO) recommended seasonal malaria chemoprevention (SMC) in areas of high seasonal transmission. Though implemented since 2013, the effectiveness of SMC in Togo was never evaluated.

**Methods:**

This study concerned routine data from 2013 to 2020 mass SMC campaigns for children under five in all health facilities of three Regions of Togo. Treatment coverage, reasons for non-treatment, and SMC-attributable adverse reactions were analysed per year and treatment round. Random effect logistic models estimated SMC effectiveness per health district, year, and treatment round.

**Results:**

The overall coverage was 98% (7,971,877 doses for 8,129,668 children). Contraindication was the main reason for non-administration. Over the study period, confirmed malaria cases decreased from 11,269 (1st round of 2016) to 1395 (4th round of 2020). Only 2,398 adverse reactions were reported (prevalence: 3/10,000), but no severe Lyell syndrome or Stevens-Johnson-type skin reaction. Compared to 2016, malaria prevalence decrease was estimated at 22.6% in 2017 (p < 0.001) and 75% in 2020 (p < 0.001). SMC effectiveness ranged from 76.6% (2nd round) to 96.2% (4th round) comparison with the 1st round.

**Conclusions:**

SMC reduced significantly malaria cases among children under five. The results reassure all actors and call for effort intensification to reach the WHO goals for 2030.

**Supplementary Information:**

The online version contains supplementary material available at 10.1186/s12936-022-04434-w.

## Background

In 2019, the World Health Organization (WHO) estimated at nearly 229 million the number of malaria cases in 87 endemic countries [1]. In sub-Saharan Africa, nearly 70% of the deaths due to malaria occur in children under five [2] and, whereas the global incidence of malaria decreased by 27% between 2000 and 2015, it decreased by only 2% between 2015 and 2019, indicating a declining progress since 2015 [1].

The progress in the fight against malaria is probably due to robust strategies for prevention, diagnosis, treatment, and surveillance in endemic areas. Among these strategies, seasonal malaria chemoprevention (SMC), carried out since 2012 seems to have considerably reduced malaria morbidity and mortality, especially in children under five [3].

The effectiveness of SMC has been proven by the results of several clinical trials conducted between 2002 and 2011 in areas of high seasonal transmission of malaria in sub-Saharan Africa [4–10]. One conclusion of these studies was that “monthly administration of SMC with sulfadoxine-pyrimethamine and amodiaquine (SP + AQ) for up to three or four months during the season of high malaria transmission in children aged 3–59 months: (i) prevents about 75% of all malaria attacks; (ii) prevents about 75% of severe malaria episodes; (iii) reduces the incidence of anaemia; (iv) curbs the increase in clinical malaria during the following season; and (v) would not lead to serious adverse events” [3, 10, 11].

In Togo, malaria is still endemic in all health districts. The entire population is at risk but children under five are the most vulnerable. Currently, Togo is among the countries with stable transmission but with seasonal increases from June to October in the northern half of the country and from April to July and then from August to October in the southern half [12]. In agreement with the recommendations of the WHO, Togo has adopted the SMC as a high impact intervention to help reduce malaria morbidity and mortality in children under five. This intervention has been included in the National Malaria Control Programme (NMCP) and in the 2017–2023 National Strategic Plan.

Because of their epidemiological and pluviometry data, the northern regions of Togo (Savanes, Kara, and Centrale; 16 health districts > 60% of that area malaria cases from July to October) were the first to implement SMC. To ensure the follow-up of that strategy, the Ministry of Health and Public Hygiene (through the NMCP) has set up, since 2013, a system to collect SMC data in those regions. However, despite their availability, these data have never been analysed to assess the effectiveness of SMC as in some other countries [13–16].

This longitudinal study analysed routine data from SMC mass campaigns. It aimed to measure the effectiveness of SMC in children aged 3–59 months living in Savanes, Kara, and Centrale regions of Togo from 2013 to 2020.

## Methods

### Study type and setting

The study examined data relative to children aged 3–59 months in three regions of Togo (Savanes, Kara, and Centrale) who received SMC according to the WHO recommendation of March 2012 [3]. These data were prospectively collected by the NMCP during the seasons of heavy rainfall over a maximum of four consecutive months (or rounds) per year (July–October) between 2013 and 2020.

**Fig. 1:**
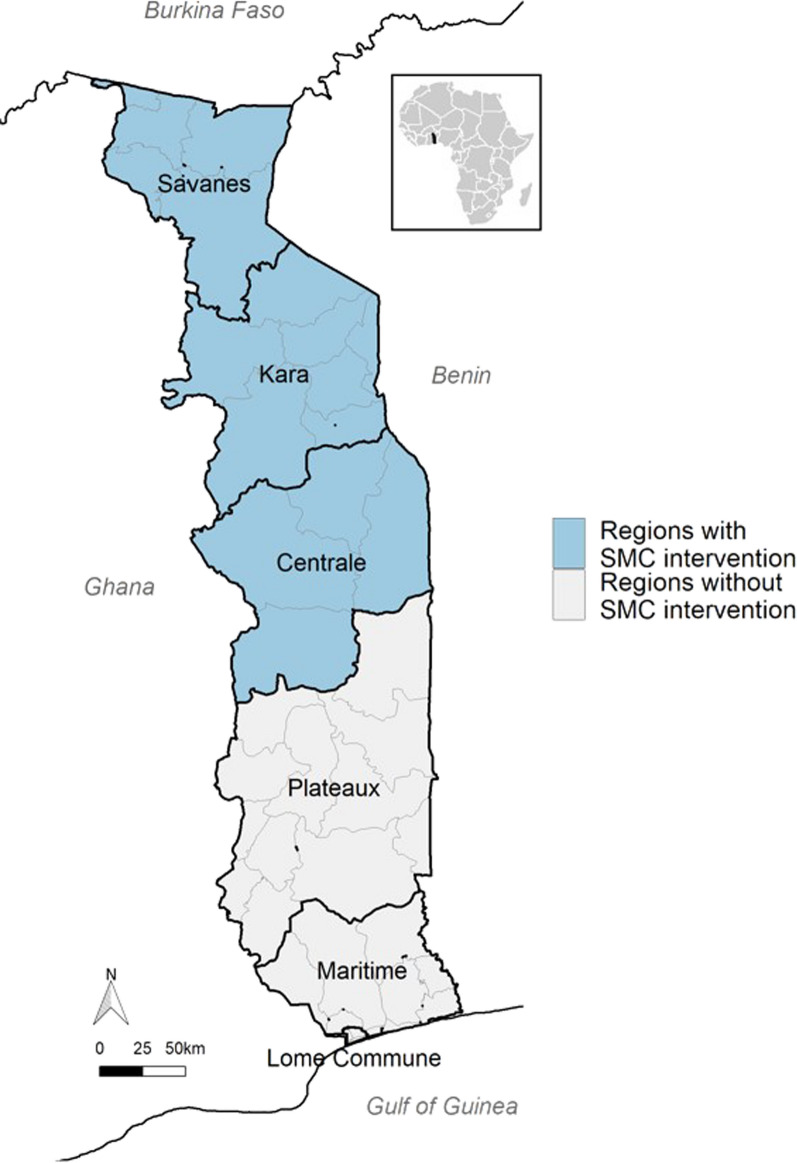
Seasonal malaria chemoprevention coverage area in Togo from 2013 to 2020 (source: National Malaria Control Programme)

The study area of 33,525 km^2^ (nearly 60% of Togo) is surrounded by Burkina Faso, Benin, Ghana, and Plateaux Region of Togo (Fig. 1). It includes three out of the six Regions of Togo, which corresponds to 16 health districts out of 44 and 324 health centers out of 987 in the whole of Togo. In 2019, the study area was home to nearly 2,829,887 people; i.e., nearly 30% of the Togolese population.

From a climatic point of view, the study area is characterized by two seasons: a dry season from November to May and a rainy season from June to October with heavy rainfall between July and September. It is during the latter period that upsurges in malaria cases are seen and that SMC is administered.

### Administration of SMC

Usually, a SMC campaign includes a maximum of four treatment rounds over four consecutive months. During each round, SMC is administered by hired community health workers (CHWs) who use two community approaches: (i) a ‘door-to-door’ approach by pairs of CHWs who visit households to identify children under five and administer the drugs; and (ii) a ‘fixed-post’ approach in health facilities to treat target children absent from home at the time of the CHWs’ visit.

A treatment round consists in administering one dose of sulfadoxine-pyrimethamine (SP) and three doses of amodiaquine (AQ) over three days. SP and the first dose of AQ are given under the supervision of a CHW; the other doses of AQ are given on the next two days by a parent or a guardian.

Before their visits to the households, the CHWs receive specific training. Besides, the NMCP ensures that SMC beneficiaries understand the objectives of the treatment. This is usually done through awareness meetings with local leaders and dissemination of messages through various public media. During an SMC campaign, the supervision is generally provided by the managers of the health facilities, the districts, the Regions, and the NMPC.

### Data collection

Sociodemographic data, administrative follow-up data, and information on adverse events were collected by 5787 CHWs from tools such as SMC cards, tally sheets, SMC treatment records, supervision forms, and adverse event count sheets. The CHWs had to report on specific sheets all adverse events occurring after treatment administration. These data were then compiled and validated by 324 health facility managers and sent to the 16 district managers. At the district level, these data were validated and transmitted to the three directors of the target Regions then to the NMCP. To improve the quality of those data and estimate the treatment coverage, the NMCP carried out community surveys at the end of each round.

### Operational definitions

Malaria prevalence is the number of children with malaria (children with positive ‘rapid diagnostic test’ (RDT) plus children already treated for malaria) divided by the number of children identified in the eligible age range.

SMC coverage is the ratio of the number of children aged 3–59 months who received all SMC doses per round, district, or region to the number of eligible children identified per round, district, or region, respectively. Previously, expected numbers of children aged 3–59 months and eligible for a SMC campaign within a given region were provided by a statistics department supervised by the Togolese Ministry of Health and actual numbers of eligible children at implementation of each SMC round (thus, year) was obtained by the CHWs.

The effectiveness of a SMC campaign was defined as the percent reduction in malaria cases in each of the two or three last months of a campaign in comparison with the first month. Precisely, over the first year of SMC, a random sample of children who had received a first round underwent parasitemia (RDT) tests at the times of administration of the second, third, and fourth round to determine the proportion of infected children one month after the end of a year’s SMC. This survey included only children whose caregivers confirmed the absence of consumption of other antimalarial drugs over each month following each round. The same survey was repeated in each of the following years. The yearly effectiveness was defined as the percent reduction in malaria cases for each year between 2017 and 2020, in comparison with year 2016. The numbers of malaria cases were obtained via health facilities and CHWs only (no external sources including pharmacies, NGOs), the proof being a positive RDT or a thick-blood-film test.

A SMC-related adverse event is any unexpected reaction in a child who received SMC treatment: vomiting, skin reactions (itching, hives), abdominal pain, somnolence, or jaundice. In Togo, cases of adverse events in children less than five are usually reported by the CHWs when administering the next round. In case an adverse event occurred soon after drug administration, the caregiver was asked either to inform the CHW (who generally lives in the same area) or visit the nearest health facility where the event is then reported on a specific sheet. Later, all adverse events are collected centrally for statistical purposes.

### Statistical analysis

The prevalence of observed malaria cases in children was modeled using a logistic mixed regression model. Separate fixed effects for ‘year’ (reference year: 2016) and ‘round’ (reference round: first round of a campaign) were estimated using indicator variables and expressed as Odds Ratios (ORs). ‘Year’ and ‘round’ effects were entered as random effects and assumed to vary randomly according to the Region, the district, and the health facility. Effectiveness was calculated as 1 − (OR_year i, round j_) with year 2016 and 1st round as references. This can be interpreted as the percent reduction of malaria prevalence. A second model was built including years 2013 to 2020.

The analyses used package *lme4* and function *glmer* in R software (R Core Team, 2017; R: A language and environment for statistical computing. R Foundation for Statistical Computing, Vienna, Austria. URL https://www.R-project.org/).

### Ethics and regulations

This study was approved in 2017 by the Ministry of Health and Public Hygiene of Togo (Memorandum No. 280/17/SPS/CAB/SG/DGPIS/DPP/DER). The data collected, compiled, and stored by the NMCP did not require obtaining informed consents.

## Results

### Participant health facilities

At the launch of SMC in Togo (Savanes Region, 2013), only 55 health facilities participated. Three years later, SMC was extended to Kara and Centrale Regions; the number of participant health facilities increased then from 301 in 2016 to 324 in 2020. Kara was the Region that participated the most (128 health facilities in 2020) and Tchaoudjo district in Centrale was the most represented district (36 health facilities yearly since 2016) (Additional file 2: Table S1).

### Target children identification

Between 2013 and 2020, the number of registered children in all three Regions increased by 4.6 times (110,354 to 510,242). Overall, Savanes had the highest number of children. In 2016, Centrale, Kara, and Savanes Centrale identified 110,067; 130,936; and 155,039; children; respectively. In 2020, those regions identified 138,743; 171,348; and 200,151; children; respectively (Additional file 3: Table S2).

### Treatment administration

Since the implementation of SMC in Togo, 7,971,877 treatments have been administered to eligible children in the three Regions. Between 2013 and 2020, the number of children treated increased by 5.7 times (from 87,918 to 502,275). In 2016, 106,273 children were treated in Centrale; 125,140 in Kara; and 150,815 in Savanes. In 2020, the same Regions treated 135,861; 168,017; and 198,397; children; respectively (Table 1).

**Table 1:** Absolute numbers of children who received seasonal malaria chemoprevention per district and region, Togo, 2013–2020

Region + district	2013 rounds			2014 rounds			2016 rounds			2017 rounds				2018 rounds			2019 rounds			2020 rounds				Total
	1	2	3	1	2	3	1	2	3	1	2	3	4	1	2	3	1	2	3	1	2	3	4	
*CENTRALE*	NA	NA	NA	NA	NA	NA																		
Blitta	–	–	–	–	–	–	23,282	24,425	24,893	24,947	25,722	25,809	26,336	25,052	26,425	26,540	28,272	29,277	29,444	28,679	29,862	30,876	31,511	461,352
Sotouboua	–	–	–	–	–	–	25,095	27,084	28,138	27,359	28,869	28,910	28,942	27,036	28,922	29,376	28,783	30,425	30,456	28,713	30,743	31,684	31,841	492,376
Tchamba	–	–	–	–	–	–	23,576	26,040	27,323	25,180	25,703	26,240	26,462	26,664	27,480	28,020	27,958	29,153	29,157	30,198	31,402	32,038	32,180	474,774
Tchaoudjo	–	–	–	–	–	–	34,320	36,000	36,717	35,263	36,518	35,379	36,432	35,075	36,192	36,544	36,398	38,050	38,161	37,744	39,683	40,492	40,329	629,297
Total	–	–	–	–	–	–	106,273	113,549	117,071	112,749	116,812	116,338	118,172	113,827	119,019	120,480	121,411	126,905	127,218	125,334	131,690	135,090	135,861	2,057,799
*KARA*	NA	NA	NA	NA	NA	NA																		
Assoli	–	–	–	–	–	–	8181	8627	9338	9368	9809	10,039	10,323	9171	9611	9674	10,721	10,198	11,021	10,792	11,928	12,518	12,298	173,617
Bassar	–	–	–	–	–	–	20,784	21,884	22,422	21,649	21,973	22,530	23,563	20,287	21,845	22,599	21,306	22,204	22,848	22,675	23,832	24,449	24,913	381,763
Binah	–	–	–	–	–	–	12,172	12,784	13,779	12,847	13,225	13,230	13,505	11,630	12,256	6491	12,809	13,690	13,640	13,676	14,168	14,509	11,875	216,286
Dankpen	–	–	–	–	–	–	25,603	28,539	29,791	29,512	29,002	28,972	29,439	27,306	28,687	29,682	27,752	29,065	29,874	29,538	31,008	31,786	31,942	497,498
Doufelgou	–	–	–	–	–	–	5526	13,966	14,521	14,138	14,505	15,090	15,411	12,710	14,364	14,639	13,165	13,882	14,285	14,327	15,042	15,645	15,952	237,168
Keran	–	–	–	–	–	–	16,771	18,885	20,626	20,675	21,146	21,723	22,001	16,344	21,244	21,822	19,836	21,029	21,609	21,443	22,719	23,137	22,676	353,686
Kozah	–	–	–	–	–	–	36,103	38,269	39,712	39,994	39,305	40,201	41,108	38,184	40,564	42,386	41,751	42,391	42,649	38,535	44,637	48,539	48,361	702,689
Total	–	–	–	–	–	–	125,140	142,954	150,189	148,183	148,965	151,785	155,350	135,632	148,571	147,293	147,340	152,459	155,926	150,986	163,334	170,583	168,017	2,562,707
*SAVANES*													NA						NA					
Cinkasse	11,271	13,139	14,028	14,573	15,798	16,022	15,053	14,629	14,814	13,331	15,944	16,385	–	15,337	15,873	16,536	14,868	15,760	–	15,920	16,814	17,201	17,466	320,762
Kpendjal	20,304	30,948	32,348	35,289	36,721	38,109	31,008	32,964	34,201	27,505	34,698	35,849	–	33,379	36,423	38,843	35,295	35,854	–	36,831	38,602	40,509	40,666	726,346
Oti	NA	NA	NA	36,698	37,335	40,034	34,363	37,263	38,684	21,253	37,643	38,690	–	39,639	39,002	14,852	40,565	42,362	–	44,960	47,480	48,665	49,492	688,980
Tandjoare	16,026	20,798	7605	21,814	22,888	23,268	20,046	21,245	21,664	8840	21,210	23,390	–	23,319	23,801	25,127	24,039	25,155	–	24,544	25,186	25,632	25,684	451,281
Tone	40,317	50,205	50,050	55,283	56,872	59,768	50,345	52,198	53,110	51,733	54,669	56,433	–	53,137	51,488	56,979	58,495	60,393	–	60,458	63,285	63,695	65,089	1,164,002
Total	87,918	115,090	104,031	163,657	169,614	177,201	150,815	158,299	162,473	122,662	164,164	170,747	–	164,811	166,587	152,337	173,262	179,524	–	182,713	191,367	195,702	198,397	3,351,371
Overall total	87,918	115,090	104,031	163,657	169,614	177,201	382,228	414,802	429,733	383,594	429,941	438,870	273,522	414,270	434,177	420,110	442,013	458,888	283,144	459,033	486,391	501,375	502,275	7,971,877

*NA* districts where seasonal malaria chemoprevention was not used yet or could not be used for logistic reasons

### SMC coverage

From 2013 to 2020, the SMC coverage rate exceeded the objective of the WHO (at least 95%); it was higher than 98% in all regions. However, there were some differences between districts; e.g., 99% in Blitta, Kéran, and Oti vs. 96% in Kpendjal. The lowest coverage of 80% was observed in Savanes in 2013 (the year SMC was launched in Togo) because several health facilities experienced shortage of drugs (Table 2).

**Table 2:** Seasonal malaria chemoprevention coverage rates per district and region, Togo, 2013–2020

Region and district	2013 rounds			2014 rounds			2016 rounds			2017 rounds				2018 rounds			2019 rounds			2020 rounds				Total
	1	2	3	1	2	3	1	2	3	1	2	3	4	1	2	3	1	2	3	1	2	3	4	
*CENTRALE*	NA	NA	NA	NA	NA	NA																		
Blitta	–	–	–	–	–	–	97.7	98.4	98.6	98.4	99.0	99.0	99.6	98.1	99.3	98.8	99.2	99.1	99.0	99.3	99.4	99.1	98.5	98.9
Sotouboua	–	–	–	–	–	–	95.5	97.6	98.0	96.9	98.2	98.8	99.2	97.6	99.1	98.8	98.3	98.6	97.9	98.3	98.6	98.1	98.3	98.1
Tchamba	–	–	–	–	–	–	96.4	97.9	97.5	97.6	98.4	98.5	98.5	97.5	98.6	98.4	98.3	98.0	97.5	99.1	98.2	97.5	97.2	98.0
Tchaoudjo	–	–	–	–	–	–	96.7	96.8	96.7	98.2	98.1	95.8	96.7	98.6	98.6	98.1	99.0	98.6	97.9	99.2	99.0	98.3	97.7	97.9
Total	–	–	–	–	–	–	96.6	97.6	97.6	97.8	98.4	97.9	98.3	98.0	98.9	98.5	98.7	98.5	98.1	99.0	98.8	98.3	97.9	98.2
*KARA*	NA	NA	NA	NA	NA	NA																		
Assoli	–	–	–	–	–	–	95.8	97.8	99.2	98.0	97.3	96.8	97.1	99.2	98.8	99.0	99.1	99.1	97.9	99.2	98.9	98.8	98.1	98.3
Bassar	–	–	–	–	–	–	96.0	97.6	98.8	97.3	98.2	98.1	98.1	98.1	98.3	98.0	97.4	98.1	97.8	97.9	98.3	98.2	98.2	97.9
Binah	–	–	–	–	–	–	94.7	98.4	99.5	95.3	97.5	96.6	97.3	95.5	96.9	97.7	96.1	97.6	97.4	97.8	97.6	97.2	97.6	97.1
Dankpen	–	–	–	–	–	–	93.4	97.6	98.6	97.1	97.8	98.0	98.9	98.4	98.7	99.1	98.4	97.8	97.9	98.5	98.1	98.2	98.0	97.9
Doufelgou	–	–	–	–	–	–	96.6	98.8	99.5	96.7	98.2	97.8	98.4	98.5	99.1	99.1	98.2	98.3	96.8	98.6	98.0	97.8	96.9	98.1
Keran	–	–	–	–	–	–	95.8	98.7	99.6	97.5	99.4	99.5	99.7	99.1	99.8	99.7	98.2	99.5	99.5	98.4	99.2	99.2	98.9	99.0
Kozah	–	–	–	–	–	–	96.9	98.5	99.2	97.7	97.8	97.7	98.3	98.3	98.4	98.8	98.1	98.1	97.4	98.1	98.3	98.1	98.1	98.1
Total	–	–	–	–	–	–	95.6	98.2	99.1	97.2	98.1	97.9	98.4	98.2	98.6	98.9	98.0	98.3	97.8	98.3	98.4	98.2	98.1	98.1
*SAVANES*													NA						NA					
Cinkasse	86.4	99.1	99.9	97.9	99.1	98.8	97.8	98.6	99.2	95.5	98.6	98.9	–	97.6	97.8	98.5	98.3	98.7	–	98.7	98.8	98.4	98.8	98.0
Kpendjal	67.3	97.5	99.6	96.1	97.1	97.8	96.0	98.1	99.3	92.5	98.1	98.7	–	96.8	98.3	98.6	97.8	98.5	–	97.4	98.2	98.7	98.7	96.5
Oti	NA	NA	NA	93.3	99.1	99.3	98.6	99.5	99.8	98.4	99.8	99.8	–	99.4	99.8	99.9	99.8	99.9	–	99.5	99.4	99.4	99.4	99.1
Tandjoare	84.5	96.9	99.2	96.7	98.4	98.9	96.6	98.9	99.2	97.3	99.1	99.4	–	98.7	98.8	99.3	98.4	99.6	–	98.9	99.5	99.6	99.3	98.1
Tone	83.6	96.7	99.3	97.4	99.2	98.9	97.3	99.0	99.5	97.8	99.2	99.2	–	98.7	99.2	99.1	99.0	99.2	–	99.0	98.4	99.1	99.2	98.1
Total	79.7	97.2	99.5	96.1	98.6	98.8	97.3	98.9	99.4	96.4	99.0	99.2	–	98.4	98.9	99.0	98.8	99.2	–	98.8	98.8	99.1	99.1	98.0
Overall total	79.7	97.2	99.5	96.1	98.6	98.8	96.5	98.3	98.8	97.1	98.5	98.4	98.4	98.2	98.8	98.8	98.5	98.7	97.9	98.7	98.7	98.6	98.4	98.1

*NA* districts where seasonal malaria chemoprevention was not used yet or could not be used for logistic reasons

### Reasons for not administering SMC

Children who did not receive SMC represented 1.9% of all children identified in the study area. The main reasons were febrile state and positive RDT or intake of artemether-lumefantrine tablets (Coartem^®^ or Riamet^®^) (35%), absence from home at the time of the CHW’s visit (28.9%), current malaria treatment (10.7%), and current cotrimoxazole intake (6.7%). The reason could not be found in 15 2% of cases (Additional file 4: Table S3).

### Side effects

On 7,971,877 target children treated from 2013 to 2020, the safety reports indicated 2,398 adverse reactions, which is an overall prevalence of 3 per 10,000 children treated. The most frequent adverse events were: vomiting (66.1%), drowsiness (11.2%), rash (10.2%), and abdominal pain (5.2%). No severe skin reaction (such as Stevens-Johnson or Lyell syndrome) was reported (Additional file 5: Table S4).

### SMC effectiveness

The study showed significant reductions in malaria cases both at round and year levels.

The number of confirmed malaria cases among identified target children dropped from 11,267 cases (2.8% prevalence) in the 1st round of 2016 to 1395 cases (0.3% prevalence) in the 4th round of 2020 (Additional file 6: Table S5). With 2016 as reference, the percent reduction of malaria prevalence was estimated at 22.6% in 2017 (p < 0.001) and reached 75% in 2020 (p < 0.001) (Table 3). The effectiveness of SMC ranged from 76.6% for the second round to 96.2% for the fourth round in comparison with the first round.

**Table 3:** Effectiveness of seasonal malaria chemoprevention relative to reference year 2016 and round 1 in 324 health facilities of 16 districts of Togo, 2016–2020

	Odds Ratio	Effectiveness % (95% CI)	p-value
*Year*			
2017	0.77	22.6 (17.5–27.3)	< 0.001
2018	0.53	46.6 (39.8–52.7)	< 0.001
2019	0.44	55.9 (47.3–63.1)	< 0.001
2020	0.25	75.0 (68.3–80.3)	< 0.001
*Round*			
2	0.23	76.6 (72.4–80.2)	< 0.001
3	0.12	88.0 (86.1–89.6)	< 0.001
4	0.04	96.2 (95.3–97.0)	< 0.001

*CI* confidence interval

**Fig. 2:**
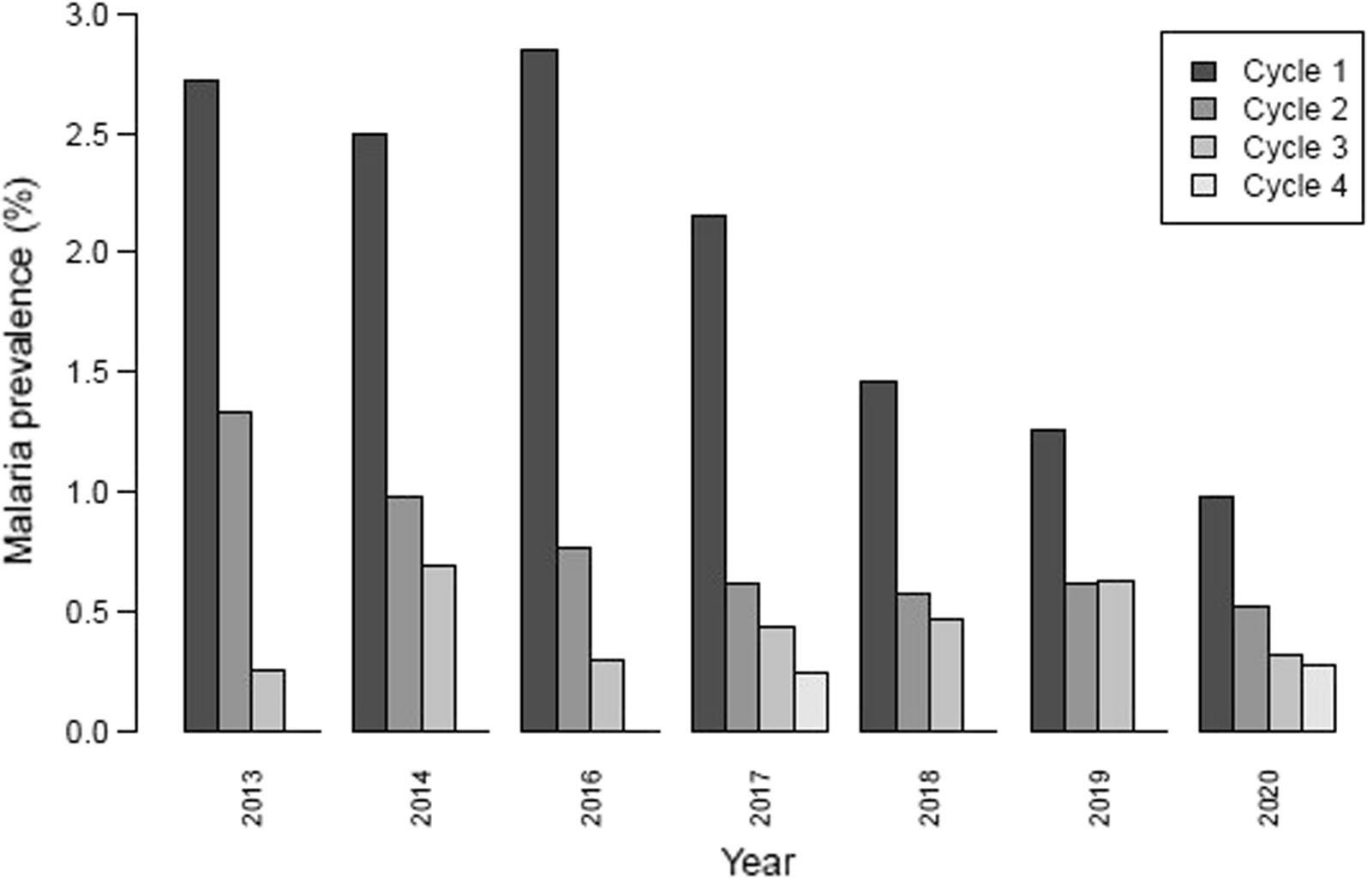
Changes in malaria prevalence from 2013 to 2020 by year and round in whole Togo. Prevalence is defined as the number of children with malaria (children with positive ‘rapid diagnostic test’ (RDT) plus children already treated for malaria) divided by the number of children identified in the eligible age range

**Fig. 3:**
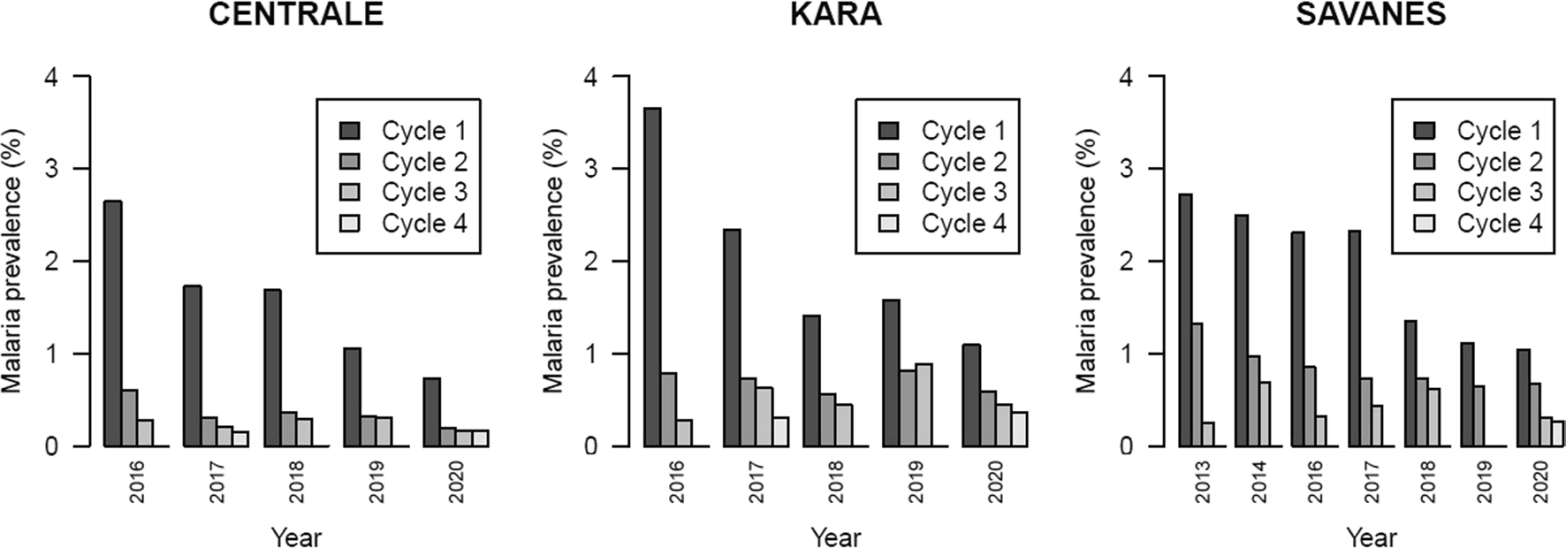
Changes in malaria prevalence (%) by region, year and round in Togo from 2013 to 2020 in Savanes region and from 2016 to 2020 in Central and Kara regions. Prevalence is defined as the number of children with malaria (children with positive ‘rapid diagnostic test’ (RDT) plus children already treated for malaria) divided by the number of children identified in the eligible age range

According to the years of use, the effectiveness of SMC ranged from 1.5% in 2014 to 69.7% in 2020 in comparison with year 2013 (Table 4). According to the rounds, that effectiveness ranged from 68.2% for the second round to 96% for the 4th round in comparison with first round. There was also a heterogeneity in effectiveness between districts (e.g., Cinkassé, Kpendjal, Tandjoaré, Oti, Tone), or regions (Fig. 2, Additional file 6: Table S5, Additional file 1: Fig. S1). One interesting result is that rebounds were observed each year at the 1st round of SMC (Fig. 3).

**Table 4:** Effectiveness of seasonal malaria chemoprevention relative to reference year 2013 and round 1 in 324 health facilities of 16 districts of Togo, 2013–2020

	Odds Ratios	Effectiveness % (95% CI)	p-value
*Year*			
2014	0.98	1.49 (− 6.1 to + 8.6)	0.693
2016	0.74	26.20 (10.3–39.3)	0.002
2017	0.66	34.56 (15.3–49.4)	0.001
2018	0.47	52.96 (35.2–65.9)	< 0.001
2019	0.35	65.01 (48.6–76.2)	< 0.001
2020	0.30	69.73 (52.7–80.6)	< 0.001
*Round*			
2	0.32	68.21 (56.3–76.9)	< 0.001
3	0.14	86.07 (83.0–88.6)	< 0.001
4	0.04	96.04 (93.6–97.5)	< 0.001

*CI* confidence interval

## Discussion

Evaluating the effectiveness of strategies of fight against malaria allow health authorities to know the real impact of each strategy and make decisions relative to its sustainability and generalizability. The present results from Togo report reductions in malaria cases in children less than five wherever (all districts and all health regions) and whenever (all rounds and all years) SMC strategy was used.

Before 2013, the year of implementation of SMC in Togo (2013), there were frequent upsurges in malaria cases during the rainy seasons, especially among children under five. Indeed, in a recent study on malaria-related morbidity and mortality trends in Togo from 2008 to 2017 [12], morbidity tended to rise throughout Togo despite intensifications of various strategies and interventions. The present study shows that, since the start of SMC, there were only occasional fluctuations in malaria cases suggesting a decrease in the prevalence of malaria and a success of SMC. This is in agreement with: (i) fewer admissions of children under five to hospital for malaria during the rainy seasons (NMCP internal reports); and (ii) a rebound in malaria cases before each SMC yearly campaign.

Currently, there is no well-established way to estimate SMC effectiveness. The decrease in the prevalence of malaria cases as a function of time may be interpreted as an indication of SMC effectiveness, especially that, in the present study: (i) the coverage rate over time remained rather stable; (ii) SMC drug quality was ascertained by the PNLP; (iii) children identification system did not change; and, (iv) several major determinants of malaria transmission (incl. rainfall patterns, antivector fight) remained practically the same in SMC-targeted areas. Nevertheless, another way of estimating SMC effectiveness would consist in examining malaria incidence among non-targeted children (aged 6–10 years) per round and year. This is worth being implemented and its results compared with those of the current approach.

The specialized literature does not mention yet studies of SMC effectiveness outside Africa. In Africa, most studies on SMC were carried out in sub-Saharan West and Central Africa [11, 17–21] and concerned mostly limited areas (few villages or few districts) [11, 14, 15, 22–26]. The present study is most probably the first in Togo to evaluate the effectiveness of SMC strategy in children since its inception in that country, with one major asset that it was exhaustive over all health facilities of all districts where SMC was used. Apparently, a comparable exhaustiveness was seen only in Nigeria and Ghana [27, 28]. Here, it is worth mentioning that, among the strategies the Togolese NMCP has already applied to fight against malaria (insecticide nets and access to information, diagnostic, and treatment), SMC was the latest and had to be implemented according to the WHO's conditions regarding the sites (regions of high seasonal malaria transmission), the incidence (> 60% annual incidence), the morbidity (most morbidity in children), and anti-malarial drug efficacy (SP and AQ). Thus, at the time of the study, only Togolese Savanes, Kara, and Centrale regions met those criteria and were the sites of the present study. In the future, other sites will be eligible and the object of similar exhaustive investigations.

The results on SMC reported here are close to results of other studies found in the literature. Regarding morbidity and mortality, in December 2020, a large-scale observational study conducted in seven African countries (Burkina Faso, Gambia, Guinea, Mali, Niger, Nigeria, and Chad) [21] demonstrated that SMC was efficient in preventing morbidity and mortality from malaria in all seven countries. Precisely, in 2015 and 2016, nearly 12.5 and 25 million monthly SMC treatments were administered to nearly 3.6 and 7.6 million target children, respectively. The average monthly coverages were 76.4% in 2015 and 74.8% in 2016. The safety reports mentioned only 36 serious adverse reactions but no severe skin reaction. The efficacy of SMC was estimated at 88.2% over 28 days and the reduction of the number of malaria confirmed cases ranged from 25.5% (in Nigeria) to 55.2% (in Gambia). In Burkina Faso and Gambia, during the period of high transmission, SMC was respectively associated with 42.4% and 56.6% reductions in the number of deaths from malaria in hospital. Several other studies carried out in Africa have also demonstrated that SMC was well tolerated and helped reducing malaria transmission [13, 14].

In Togo, the interesting results obtained in the present study regarding malaria morbidity cannot be exclusively attributed to SMC; they result most probably from a combination of several concurrent preventive and curative interventions. Showing a SMC-specific efficiency requires conducting a clinical trial or a case–control study. Nevertheless, one asset of the present study is that 7,971,877 treatments (SP + AQ) could be administered to a target population of 8,129,668 children identified between 2013 and 2020. This 98% average coverage rate (WHO’s minimum: 95%) is the result of a combination of several organizational factors: (i) a thorough planning; (ii) a specific training of the CHWs and other actors of the campaigns; (iii) a social mobilization to maximize the contributions of the families; (iv) a door-to-door drug administration strategy implemented by CHWs with good knowledge of their areas (and even of the families) and eager to ensure SMC of nearly all identified children; (v) the use a specific card per child to mention the administration of the doses needed and a specific register per CHW to keep all needed information on drug administration; (vi) systematic surveys after each round to check whether all identified children were actually and adequately treated; (vii) surveys to check whether implementation improvements were needed in areas where the objectives were not met; and, (viii) appropriate monitoring, supervision, and coordination of all actors. Another asset is the existence of registries for malaria cases and deaths that make it possible to assess the impact of a set of interventions (including SMC).

The interest and reliability of the present results are most probably linked with the tireless and successful efforts of several actors on the field; mainly, committed CHWs and active social partners (incl. media representatives, community leaders, religious leaders, town criers). These efforts are important to make Togo join the States committed to fighting against malaria through SMC—among other strategies. The visibility of the efforts of all people engaged in that fight (from city criers to NMCP researchers and executives) is important to continue and overcome a number of still recurrent difficulties (at least in Togo): (i) shortage of significant financial resources to cover material and personnel costs; (ii) irregular coverage of target populations over time; (iii) ensuring less SMC rounds than recommended over certain years (2013, 2014); and (iv) lack of surveillance of parasite resistance against SP or AQ [29]. Incidentally, these difficulties might have underestimated the effectiveness of SMC on some occasions and in some circumstances.

Maximizing SMC efficiency requires mobilizing all local resources, looking for additional partners, and overcoming difficulties of access to either the territories or the populations during the rainy seasons (incl. muddy roads, busy farmers). One solution is to decentralize SMC and implement it in the community by the community. An additional solution is to increase the level of adherence of the population to SMC and support the surveillance systems.

Finally, SMC could have broader benefits if it could be: (i) widened to children over five because they are an important parasite reservoir [21]; and (ii) extended to areas less subject to malaria seasonal transmission.

## Conclusion

The implementation of SMC in part of Togo between 2013 and 2020 has contributed to a reduction of malaria morbidity through a very high coverage rate that should be carefully monitored, sustained, and even increased and extended. This requires finding additional economic international partners of the NMCP and involving more national actors from the government, the private sector, and the civil society.

## Supplementary Information

**Additional file 1: Figure S1.** Heterogeneity of yearly and cycle seasonal malaria chemoprevention effects in the districts of Togo from 2016 to 2020. Deviation of each district from the average is represented for yearly and cycle effects. Districts below line 0 (Blitta, Oti, Sotouboua, Tchamba, and Tchaoudjo) showed better effectiveness of seasonal malaria chemoprevention than the average and districts above line 0 (Binah, Cinkasse, Dankpen, Kozah and Kpendjal) showed lower effectiveness.

**Additional file 2: Table S1.** Participating health facilities in seasonal malaria chemoprevention districts, Togo, 2013–2020.

**Additional file 3: Table S2.** Numbers of children identified in seasonal malaria chemoprevention zones, Togo, 2013–2020.

**Additional file 4: Table S3.** Reasons for not administering seasonal malaria chemoprevention per district, Togo, 2013–2020.

**Additional file 5: Table S4.** Side effects linked with seasonal malaria prevention drugs, Togo, 2013–2020.

**Additional file 6: Table S5.** Number of malaria cases in seasonal malaria chemoprevention zones, Togo, 2013–2020.

## Data Availability

Not applicable.
