# Supplementary material for: Effectiveness of seasonal malaria chemoprevention in three regions of Togo: a population-based longitudinal study from 2013 to 2020
Source: Malar J. 2022 Dec 31;21:400. doi: 10.1186/s12936-022-04434-w (PMC9804945; doi:10.1186/s12936-022-04434-w)
Supplement: Supplementary file 1 — Additional file 1: Figure S1. Heterogeneity of yearly and cycle seasonal malaria chemoprevention effects in the districts of Togo from 2016 to 2020. Deviation of each district from the average is represented for yearly and cycle effects. Districts below line 0 (Blitta, Oti, Sotouboua, Tchamba, and Tchaoudjo) showed better effectiveness of seasonal malaria chemoprevention than the average and districts above line 0 (Binah, Cinkasse, Dankpen, Kozah and Kpendjal) showed lower effectiveness. [file 12936_2022_4434_MOESM1_ESM.docx]

**Figure S1 – Heterogeneity of yearly and cycle seasonal malaria chemoprevention effects in the districts of Togo from 2016 to 2020. Deviation of each district from the average is represented for yearly and cycle effects. Districts below line 0 (Blitta, Oti, Sotouboua, Tchamba, and Tchaoudjo) showed better effectiveness of seasonal malaria chemoprevention than the average and districts above line 0 (Binah, Cinkasse, Dankpen, Kozah and Kpendjal) showed lower effectiveness.**
