# Supplementary material for: Effectiveness of seasonal malaria chemoprevention in three regions of Togo: a population-based longitudinal study from 2013 to 2020
Source: Malar J. 2022 Dec 31;21:400. doi: 10.1186/s12936-022-04434-w (PMC9804945; doi:10.1186/s12936-022-04434-w)
Supplement: Supplementary file 2 — Additional file 2: Table S1. Participating health facilities in seasonal malaria chemoprevention districts, Togo, 2013–2020. [file 12936_2022_4434_MOESM2_ESM.docx]

**Table S1 - Participating health facilities in seasonal malaria chemoprevention districts, Togo, 2013-2020.**

|  | **2013 rounds** | | |  | **2014 rounds** | | |  | **2016 rounds** | | |  | **2017 rounds** | | | |  | **2018 rounds** | | |  | **2019 rounds** | | |  | **2020 rounds** | | | |
| --- | --- | --- | --- | --- | --- | --- | --- | --- | --- | --- | --- | --- | --- | --- | --- | --- | --- | --- | --- | --- | --- | --- | --- | --- | --- | --- | --- | --- | --- |
| **Region & district** | **1** | **2** | **3** |  | **1** | **2** | **3** |  | **1** | **2** | **3** |  | **1** | **2** | **3** | **4** |  | **1** | **2** | **3** |  | **1** | **2** | **3** |  | **1** | **2** | **3** | **4** |
| *CENTRALE* | NA | NA | NA |  | NA | NA | NA |  |  |  |  |  |  |  |  |  |  |  |  |  |  |  |  |  |  |  |  |  |  |
| Blitta | .. | .. | .. |  | .. | .. | .. |  | 21 | 21 | 21 |  | 21 | 21 | 21 | 21 |  | 22 | 22 | 22 |  | 22 | 22 | 22 |  | 22 | 22 | 22 | 22 |
| Sotouboua | .. | .. | .. |  | .. | .. | .. |  | 22 | 22 | 22 |  | 23 | 23 | 23 | 23 |  | 24 | 24 | 24 |  | 25 | 25 | 25 |  | 26 | 26 | 26 | 26 |
| Tchamba | .. | .. | .. |  | .. | .. | .. |  | 18 | 18 | 18 |  | 18 | 18 | 18 | 18 |  | 18 | 18 | 18 |  | 18 | 18 | 18 |  | 21 | 21 | 21 | 21 |
| Tchaoudjo | .. | .. | .. |  | .. | .. | .. |  | 36 | 36 | 36 |  | 36 | 36 | 36 | 36 |  | 36 | 36 | 36 |  | 36 | 36 | 36 |  | 36 | 36 | 36 | 36 |
| Total | .. | .. | .. |  | .. | .. | .. |  | 97 | 97 | 97 |  | 98 | 98 | 98 | 98 |  | 100 | 100 | 100 |  | 101 | 101 | 101 |  | 105 | 105 | 105 | 105 |
| *KARA* | NA | NA | NA |  | NA | NA | NA |  |  |  |  |  |  |  |  |  |  |  |  |  |  |  |  |  |  |  |  |  |  |
| Assoli | .. | .. | .. |  | .. | .. | .. |  | 8 | 8 | 8 |  | 8 | 8 | 8 | 8 |  | 9 | 9 | 9 |  | 9 | 9 | 9 |  | 9 | 9 | 9 | 9 |
| Bassar | .. | .. | .. |  | .. | .. | .. |  | 19 | 19 | 19 |  | 19 | 19 | 19 | 19 |  | 19 | 19 | 19 |  | 19 | 19 | 19 |  | 20 | 20 | 20 | 20 |
| Binah | .. | .. | .. |  | .. | .. | .. |  | 15 | 15 | 15 |  | 16 | 16 | 16 | 16 |  | 16 | 16 | 16 |  | 16 | 16 | 16 |  | 16 | 16 | 16 | 16 |
| Dankpen | .. | .. | .. |  | .. | .. | .. |  | 16 | 16 | 16 |  | 16 | 16 | 16 | 16 |  | 18 | 18 | 18 |  | 18 | 18 | 18 |  | 18 | 18 | 18 | 18 |
| Doufelgou | .. | .. | .. |  | .. | .. | .. |  | 9 | 19 | 19 |  | 19 | 19 | 19 | 19 |  | 19 | 19 | 19 |  | 19 | 19 | 19 |  | 19 | 19 | 19 | 19 |
| Keran | .. | .. | .. |  | .. | .. | .. |  | 12 | 12 | 12 |  | 12 | 12 | 12 | 12 |  | 11 | 12 | 12 |  | 12 | 12 | 12 |  | 13 | 13 | 13 | 13 |
| Kozah | .. | .. | .. |  | .. | .. | .. |  | 31 | 31 | 31 |  | 31 | 31 | 31 | 31 |  | 31 | 31 | 31 |  | 33 | 33 | 33 |  | 33 | 33 | 33 | 33 |
| Total | .. | .. | .. |  | .. | .. | .. |  | 110 | 120 | 120 |  | 121 | 121 | 121 | 121 |  | 123 | 124 | 124 |  | 126 | 126 | 126 |  | 128 | 128 | 128 | 128 |
| *SAVANES* |  |  |  |  |  |  |  |  |  |  |  |  |  |  |  | NA |  |  |  |  |  |  |  | NA |  |  |  |  |  |
| Cinkasse | 7 | 7 | 7 |  | 7 | 7 | 7 |  | 7 | 7 | 7 |  | 7 | 7 | 7 | .. |  | 7 | 7 | 7 |  | 7 | 7 | .. |  | 7 | 7 | 7 | 7 |
| Kpendjal | 14 | 14 | 14 |  | 14 | 14 | 14 |  | 14 | 14 | 14 |  | 14 | 14 | 14 | .. |  | 14 | 14 | 14 |  | 16 | 16 | .. |  | 16 | 16 | 16 | 16 |
| Oti | NA | NA | NA |  | 19 | 19 | 19 |  | 19 | 19 | 19 |  | 22 | 22 | 22 | .. |  | 22 | 22 | 22 |  | 22 | 22 | .. |  | 22 | 23 | 23 | 23 |
| Tandjoare | 15 | 15 | 15 |  | 16 | 16 | 16 |  | 17 | 17 | 17 |  | 13 | 17 | 17 | .. |  | 17 | 17 | 17 |  | 17 | 17 | .. |  | 17 | 17 | 17 | 17 |
| Tone | 19 | 19 | 18 |  | 19 | 19 | 19 |  | 27 | 27 | 27 |  | 27 | 27 | 27 | .. |  | 27 | 27 | 27 |  | 27 | 27 | .. |  | 27 | 28 | 28 | 28 |
| Total | 55 | 55 | 54 |  | 75 | 75 | 75 |  | 84 | 84 | 84 |  | 83 | 87 | 87 | .. |  | 87 | 87 | 87 |  | 89 | 89 | .. |  | 89 | 91 | 91 | 91 |
| Overall total | 55 | 55 | 54 |  | 75 | 75 | 75 |  | 291 | 301 | 301 |  | 302 | 306 | 306 | 219 |  | 310 | 311 | 311 |  | 316 | 316 | 227 |  | 322 | 324 | 324 | 324 |

NA: Districts where SMC was not used yet or could not be used for logistic reasons.
