# Supplementary material for: Effectiveness of seasonal malaria chemoprevention in three regions of Togo: a population-based longitudinal study from 2013 to 2020
Source: Malar J. 2022 Dec 31;21:400. doi: 10.1186/s12936-022-04434-w (PMC9804945; doi:10.1186/s12936-022-04434-w)
Supplement: Supplementary file 3 — Additional file 3: Table S2. Numbers of children identified in seasonal malaria chemoprevention zones, Togo, 2013–2020. [file 12936_2022_4434_MOESM3_ESM.docx]

**Table S2 - Numbers of children identified in seasonal malaria chemoprevention zones, Togo, 2013-2020.**

|  | **2013 rounds** | | |  | **2014 rounds** | | |  | **2016 rounds** | | |  | **2017 rounds** | | | |  | **2018 rounds** | | |  | **2019 rounds** | | |  | **2020 rounds** | | | |  |
| --- | --- | --- | --- | --- | --- | --- | --- | --- | --- | --- | --- | --- | --- | --- | --- | --- | --- | --- | --- | --- | --- | --- | --- | --- | --- | --- | --- | --- | --- | --- |
| **Region & district** | **1** | **2** | **3** |  | **1** | **2** | **3** |  | **1** | **2** | **3** |  | **1** | **2** | **3** | **4** |  | **1** | **2** | **3** |  | **1** | **2** | **3** |  | **1** | **2** | **3** | **4** |  |
| *CENTRALE* | NA | NA | NA |  | NA | NA | NA |  |  |  |  |  |  |  |  |  |  |  |  |  |  |  |  |  |  |  |  |  |  |  |
| Blitta | .. | .. | .. |  | .. | .. | .. |  | 23834 | 24830 | 25251 |  | 25340 | 25971 | 26071 | 26446 |  | 25526 | 26598 | 26857 |  | 28512 | 29550 | 29743 |  | 28881 | 30032 | 31161 | 31987 | 466590 |
| Sotouboua | .. | .. | .. |  | .. | .. | .. |  | 26268 | 27743 | 28716 |  | 28225 | 29384 | 29261 | 29166 |  | 27710 | 29186 | 29732 |  | 29294 | 30868 | 31123 |  | 29201 | 31175 | 32304 | 32380 | 501736 |
| Tchamba | .. | .. | .. |  | .. | .. | .. |  | 24458 | 26607 | 28021 |  | 25810 | 26125 | 26631 | 26876 |  | 27335 | 27865 | 28472 |  | 28437 | 29758 | 29895 |  | 30462 | 31963 | 32853 | 33104 | 484672 |
| Tchaoudjo | .. | .. | .. |  | .. | .. | .. |  | 35507 | 37195 | 37966 |  | 35914 | 37211 | 36926 | 37686 |  | 35557 | 36705 | 37244 |  | 36774 | 38598 | 38986 |  | 38065 | 40068 | 41172 | 41272 | 642846 |
| Total | .. | .. | .. |  | .. | .. | .. |  | 110067 | 116375 | 119954 |  | 115289 | 118691 | 118889 | 120174 |  | 116128 | 120354 | 122305 |  | 123017 | 128774 | 129747 |  | 126609 | 133238 | 137490 | 138743 | 2095844 |
| *KARA* | NA | NA | NA |  | NA | NA | NA |  |  |  |  |  |  |  |  |  |  |  |  |  |  |  |  |  |  |  |  |  |  |  |
| Assoli | .. | .. | .. |  | .. | .. | .. |  | 8538 | 8817 | 9414 |  | 9558 | 10085 | 10374 | 10636 |  | 9248 | 9732 | 9775 |  | 10815 | 10291 | 11261 |  | 10883 | 12062 | 12671 | 12533 | 176693 |
| Bassar | .. | .. | .. |  | .. | .. | .. |  | 21646 | 22413 | 22685 |  | 22247 | 22373 | 22977 | 24020 |  | 20674 | 22225 | 23060 |  | 21874 | 22634 | 23359 |  | 23173 | 24234 | 24906 | 25382 | 389882 |
| Binah | .. | .. | .. |  | .. | .. | .. |  | 12860 | 12998 | 13846 |  | 13487 | 13559 | 13697 | 13873 |  | 12172 | 12645 | 6646 |  | 13325 | 14029 | 14005 |  | 13984 | 14510 | 14923 | 12165 | 222724 |
| Dankpen | .. | .. | .. |  | .. | .. | .. |  | 27400 | 29242 | 30210 |  | 30388 | 29665 | 29572 | 29754 |  | 27764 | 29075 | 29953 |  | 28216 | 29712 | 30516 |  | 29994 | 31619 | 32361 | 32579 | 508020 |
| Doufelgou | .. | .. | .. |  | .. | .. | .. |  | 5723 | 14137 | 14594 |  | 14621 | 14770 | 15431 | 15659 |  | 12903 | 14489 | 14766 |  | 13401 | 14118 | 14758 |  | 14524 | 15343 | 16005 | 16465 | 241707 |
| Keran | .. | .. | .. |  | .. | .. | .. |  | 17515 | 19141 | 20714 |  | 21216 | 21282 | 21829 | 22062 |  | 16488 | 21294 | 21882 |  | 20194 | 21129 | 21716 |  | 21800 | 22892 | 23335 | 22939 | 357428 |
| Kozah | .. | .. | .. |  | .. | .. | .. |  | 37254 | 38869 | 40051 |  | 40940 | 40190 | 41168 | 41840 |  | 38832 | 41213 | 42910 |  | 42573 | 43225 | 43783 |  | 39289 | 45396 | 49495 | 49285 | 716313 |
| Total | .. | .. | .. |  | .. | .. | .. |  | 130936 | 145617 | 151514 |  | 152457 | 151924 | 155048 | 157844 |  | 138081 | 150673 | 148992 |  | 150398 | 155138 | 159398 |  | 153647 | 166056 | 173696 | 171348 | 2612767 |
| *SAVANES* |  |  |  |  |  |  |  |  |  |  |  |  |  |  |  | NA |  |  |  |  |  |  |  | NA |  |  |  |  |  |  |
| Cinkasse | 13046 | 13256 | 14049 |  | 14888 | 15945 | 16212 |  | 15396 | 14836 | 14929 |  | 13966 | 16168 | 16561 | .. |  | 15707 | 16225 | 16786 |  | 15120 | 15970 | .. |  | 16132 | 17026 | 17479 | 17681 | 327378 |
| Kpendjal | 30149 | 31737 | 32470 |  | 36703 | 37802 | 38960 |  | 32305 | 33591 | 34450 |  | 29742 | 35384 | 36304 | .. |  | 34472 | 37048 | 39389 |  | 36106 | 36406 | .. |  | 37807 | 39308 | 41055 | 41189 | 752377 |
| Oti | NA | NA | NA |  | 39322 | 37671 | 40317 |  | 34855 | 37452 | 38760 |  | 21600 | 37718 | 38784 | .. |  | 39867 | 39087 | 14872 |  | 40663 | 42417 | .. |  | 45170 | 47746 | 48948 | 49812 | 695061 |
| Tandjoare | 18958 | 21467 | 7670 |  | 22548 | 23257 | 23520 |  | 20747 | 21492 | 21839 |  | 9081 | 21394 | 23522 | .. |  | 23636 | 24087 | 25316 |  | 24438 | 25254 | .. |  | 24810 | 25313 | 25736 | 25865 | 459950 |
| Tone | 48201 | 51926 | 50410 |  | 56771 | 57356 | 60409 |  | 51736 | 52716 | 53400 |  | 52889 | 55126 | 56898 | .. |  | 53814 | 51925 | 57505 |  | 59101 | 60856 | .. |  | 61043 | 64341 | 64264 | 65604 | 1186291 |
| Total | 110354 | 118386 | 104599 |  | 170232 | 172031 | 179418 |  | 155039 | 160087 | 163378 |  | 127278 | 165790 | 172069 | .. |  | 167496 | 168372 | 153868 |  | 175428 | 180903 | .. |  | 184962 | 193734 | 197482 | 200151 | 3421057 |
| Overall total | 110354 | 118386 | 104599 |  | 170232 | 172031 | 179418 |  | 396042 | 422079 | 434846 |  | 395024 | 436405 | 446006 | 278018 |  | 421705 | 439399 | 425165 |  | 448843 | 464815 | 289145 |  | 465218 | 493028 | 508668 | 510242 | 8129668 |

NA: Districts where SMC was not used yet or could not be used for logistic reasons.
