# Supplementary material for: Effectiveness of seasonal malaria chemoprevention in three regions of Togo: a population-based longitudinal study from 2013 to 2020
Source: Malar J. 2022 Dec 31;21:400. doi: 10.1186/s12936-022-04434-w (PMC9804945; doi:10.1186/s12936-022-04434-w)
Supplement: Supplementary file 4 — Additional file 4: Table S3. Reasons for not administering seasonal malaria chemoprevention per district, Togo, 2013–2020. [file 12936_2022_4434_MOESM4_ESM.docx]

**Table S3 - Reasons for not administering seasonal malaria chemoprevention per district, Togo, 2013-2020.**

| **Region & district** | **Allergy** | **Severe disease** | **Refusal** | **Under CTX** | **Absent** | **Positive RDT + under ACT** | **Under malaria treatment** | **Unknown reason** | **Total** | **Percent of target children** |
| --- | --- | --- | --- | --- | --- | --- | --- | --- | --- | --- |
| *CENTRALE* |  |  |  |  |  |  |  |  |  |  |
| Blitta | 67 (1.3) | 150 (2.9) | 56 (1.1) | 309 (5.9) | 2282 (43.6) | 2024 (38.6) | 257 (4.9) | 93 (1.8) | 5238 | 1.1 |
| Sotouboua | 27 (0.3) | 428 (4.6) | 56 (0.6) | 629 (6.7) | 4399 (47) | 3330 (35.6) | 358 (3.8) | 133 (1.4) | 9360 | 1.9 |
| Tchamba | 98 (1) | 146 (1.5) | 15 (0.2) | 327 (3.3) | 5376 (54.3) | 3043 (30.7) | 805 (8.1) | 88 (0.9) | 9898 | 2 |
| Tchaoudjo | 171 (1.3) | 312 (2.3) | 70 (0.5) | 740 (5.5) | 8883 (65.6) | 2924 (21.6) | 442 (3.3) | 7 (0.1) | 13549 | 2.1 |
| Total | 363 (1) | 1036 (2.7) | 197 (0.5) | 2005 (5.3) | 20940 (55) | 11321 (29.8) | 1862 (4.9) | 321 (0.8) | 38045 | 1.8 |
| *KARA* |  |  |  |  |  |  |  |  |  |  |
| Assoli | 72 (2.3) | 81 (2.6) | 6 (0.2) | 267 (8.7) | 1583 (51.5) | 944 (30.7) | 97 (3.2) | 26 (0.8) | 3076 | 1.7 |
| Bassar | 24 (0.3) | 169 (2.1) | 23 (0.3) | 791 (9.7) | 3027 (37.3) | 1920 (23.6) | 2159 (26.6) | 6 (0.1) | 8119 | 2.1 |
| Binah | 37 (0.6) | 134 (2.1) | 9 (0.1) | 322 (5) | 1917 (29.8) | 2473 (38.4) | 1512 (23.5) | 34 (0.5) | 6438 | 2.9 |
| Dankpen | 13 (0.1) | 255 (2.4) | 19 (0.2) | 504 (4.8) | 3083 (29.3) | 3546 (33.7) | 3022 (28.7) | 80 (0.8) | 10522 | 2.1 |
| Doufelgou | 101 (2.2) | 158 (3.5) | 6 (0.1) | 326 (7.2) | 2358 (51.9) | 1386 (30.5) | 187 (4.1) | 17 (0.4) | 4539 | 1.9 |
| Keran | 24 (0.6) | 168 (4.5) | 1 (0) | 813 (21.7) | 458 (12.2) | 1844 (49.3) | 427 (11.4) | 7 (0.2) | 3742 | 1 |
| Kozah | 368 (2.7) | 390 (2.9) | 117 (0.9) | 1597 (11.7) | 5765 (42.3) | 3972 (29.2) | 1409 (10.3) | 6 (0) | 13624 | 1.9 |
| Total | 639 (1.3) | 1355 (2.7) | 181 (0.4) | 4620 (9.2) | 18191 (36.3) | 16085 (32.1) | 8813 (17.6) | 176 (0.4) | 50060 | 1.9 |
| *SAVANES* |  |  |  |  |  |  |  |  |  |  |
| Cinkasse | 72 (1.1) | 188 (2.8) | 2 (0) | 240 (3.6) | 945 (14.3) | 2727 (41.2) | 668 (10.1) | 1774 (26.8) | 6616 | 2 |
| Kpendjal | 276 (1.1) | 132 (0.5) | 13 (0) | 1212 (4.7) | 1406 (5.4) | 11046 (42.4) | 2211 (8.5) | 9735 (37.4) | 26031 | 3.5 |
| Oti | 64 (1.1) | 197 (3.2) | 6 (0.1) | 454 (7.5) | 977 (16.1) | 2515 (41.4) | 349 (5.7) | 1519 (25) | 6081 | 0.9 |
| Tandjoare | 145 (1.7) | 124 (1.4) | 2 (0) | 562 (6.5) | 312 (3.6) | 4798 (55.3) | 298 (3.4) | 2428 (28) | 8669 | 1.9 |
| Tone | 361 (1.6) | 258 (1.2) | 40 (0.2) | 1401 (6.3) | 2772 (12.4) | 6787 (30.4) | 2700 (12.1) | 7970 (35.8) | 22289 | 1.9 |
| Total | 918 (1.3) | 899 (1.3) | 63 (0.1) | 3869 (5.6) | 6412 (9.2) | 27873 (40) | 6226 (8.9) | 23426 (33.6) | 69686 | 2 |
| Overall total | 1920 (1.2) | 3290 (2.1) | 441 (0.3) | 10494 (6.7) | 45543 (28.9) | 55279 (35) | 16901 (10.7) | 23923 (15.2) | 157791 | 1.9 |

CTX: Cotrimoxazole – RDT: Rapid diagnostic test for malaria- ACT: Artemisinin-based combination therapy; here, artemisinin + lumefantrine.
