# Supplementary material for: Effectiveness of seasonal malaria chemoprevention in three regions of Togo: a population-based longitudinal study from 2013 to 2020
Source: Malar J. 2022 Dec 31;21:400. doi: 10.1186/s12936-022-04434-w (PMC9804945; doi:10.1186/s12936-022-04434-w)
Supplement: Supplementary file 5 — Additional file 5: Table S4. Side effects linked with seasonal malaria prevention drugs, Togo, 2013–2020. [file 12936_2022_4434_MOESM5_ESM.docx]

**Table S4 - Side effects linked with seasonal malaria prevention drugs, Togo, 2013-2020.**

| **Region & district** | **Vomiting** | **Skin reactions** | **Drowsiness** | **Stomach ache** | **Other** | **Total** | **Rate per 1000** |
| --- | --- | --- | --- | --- | --- | --- | --- |
| *CENTRALE* |  |  |  |  |  |  |  |
| Blitta | 108 (73) | 8 (5.4) | 12 (8.1) | 8 (5.4) | 12 (8.1) | 148 | 0.32 |
| Sotouboua | 102 (54) | 25 (13.2) | 40 (21.2) | 6 (3.2) | 16 (8.5) | 189 | 0.38 |
| Tchamba | 19 (44.2) | 8 (18.6) | 11 (25.6) | 1 (2.3) | 4 (9.3) | 43 | 0.09 |
| Tchaoudjo | 147 (73.5) | 18 (9) | 18 (9) | 8 (4) | 9 (4.5) | 200 | 0.32 |
| Total | 376 (64.8) | 59 (10.2) | 81 (14) | 23 (4) | 41 (7.1) | 580 | 0.28 |
| *KARA* |  |  |  |  |  |  |  |
| Assoli | 62 (49.6) | 6 (4.8) | 9 (7.2) | 16 (12.8) | 32 (25.6) | 125 | 0.72 |
| Bassar | 110 (67.1) | 7 (4.3) | 10 (6.1) | 2 (1.2) | 35 (21.3) | 164 | 0.43 |
| Binah | 95 (86.4) | 4 (3.6) | 8 (7.3) | 1 (0.9) | 2 (1.8) | 110 | 0.51 |
| Dankpen | 21 (70) | 4 (13.3) | 3 (10) | 1 (3.3) | 1 (3.3) | 30 | 0.06 |
| Doufelgou | 23 (59) | 3 (7.7) | 7 (17.9) | 2 (5.1) | 4 (10.3) | 39 | 0.16 |
| Keran | 16 (40) | 7 (17.5) | 9 (22.5) | 2 (5) | 6 (15) | 40 | 0.11 |
| Kozah | 253 (74.2) | 10 (2.9) | 29 (8.5) | 30 (8.8) | 19 (5.6) | 341 | 0.49 |
| Total | 580 (68.3) | 41 (4.8) | 75 (8.8) | 54 (6.4) | 99 (11.7) | 849 | 0.33 |
| *SAVANES* |  |  |  |  |  |  |  |
| Cinkasse | 19 (51.4) | 4 (10.8) | 10 (27) | 2 (5.4) | 2 (5.4) | 37 | 0.12 |
| Kpendjal | 121 (60.8) | 32 (16.1) | 32 (16.1) | 8 (4) | 6 (3) | 199 | 0.27 |
| Oti | 246 (71.9) | 47 (13.7) | 35 (10.2) | 10 (2.9) | 4 (1.2) | 342 | 0.5 |
| Tandjoare | 79 (54.5) | 40 (27.6) | 10 (6.9) | 12 (8.3) | 4 (2.8) | 145 | 0.32 |
| Tone | 164 (66.7) | 21 (8.5) | 25 (10.2) | 15 (6.1) | 21 (8.5) | 246 | 0.21 |
| Total | 629 (64.9) | 144 (14.9) | 112 (11.6) | 47 (4.9) | 37 (3.8) | 969 | 0.29 |
| Overall total | 1585 (66.1) | 244 (10.2) | 268 (11.2) | 124 (5.2) | 177 (7.4) | 2398 | 0.3 |
