# Supplementary material for: Effectiveness of seasonal malaria chemoprevention in three regions of Togo: a population-based longitudinal study from 2013 to 2020
Source: Malar J. 2022 Dec 31;21:400. doi: 10.1186/s12936-022-04434-w (PMC9804945; doi:10.1186/s12936-022-04434-w)
Supplement: Supplementary file 6 — Additional file 6: Table S5. Number of malaria cases in seasonal malaria chemoprevention zones, Togo, 2013–2020. [file 12936_2022_4434_MOESM6_ESM.docx]

**Table S5 – Number of malaria cases in seasonal malaria chemoprevention zones, Togo, 2013-2020.**

|  | **2013 rounds** | | |  | **2014 rounds** | | |  | **2016 rounds** | | |  | **2017 rounds** | | | |  | **2018 rounds** | | |  | **2019 rounds** | | |  | **2020 rounds** | | |  |
| --- | --- | --- | --- | --- | --- | --- | --- | --- | --- | --- | --- | --- | --- | --- | --- | --- | --- | --- | --- | --- | --- | --- | --- | --- | --- | --- | --- | --- | --- |
| **Region & district** | **1** | **2** | **3** |  | **1** | **2** | **3** |  | **1** | **2** | **3** |  | **1** | **2** | **3** | **4** |  | **1** | **2** | **3** |  | **1** | **2** | **3** |  | **1** | **2** | **3** | **4** |
| *CENTRALE* | NA | NA | NA |  | NA | NA | NA |  |  |  |  |  |  |  |  |  |  |  |  |  |  |  |  |  |  |  |  |  |  |
| BLITTA | .. | .. | .. |  | .. | .. | .. |  | 452 (1.9) | 137 (0.6) | 64 (0.3) |  | 338 (1.3) | 75 (0.3) | 63 (0.2) | 57 (0.2) |  | 408 (1.6) | 77 (0.3) | 65 (0.2) |  | 193 (0.7) | 58 (0.2) | 61 (0.2) |  | 155 (0.5) | 32 (0.1) | 30 (0.1) | 15 (0) |
| SOTOUBOUA | .. | .. | .. |  | .. | .. | .. |  | 837 (3.2) | 143 (0.5) | 70 (0.2) |  | 600 (2.1) | 57 (0.2) | 43 (0.1) | 38 (0.1) |  | 539 (1.9) | 115 (0.4) | 100 (0.3) |  | 398 (1.4) | 124 (0.4) | 129 (0.4) |  | 354 (1.2) | 72 (0.2) | 60 (0.2) | 60 (0.2) |
| TCHAMBA | .. | .. | .. |  | .. | .. | .. |  | 705 (2.9) | 123 (0.5) | 44 (0.2) |  | 565 (2.2) | 131 (0.5) | 74 (0.3) | 57 (0.2) |  | 618 (2.3) | 182 (0.7) | 134 (0.5) |  | 430 (1.5) | 150 (0.5) | 143 (0.5) |  | 192 (0.6) | 113 (0.4) | 102 (0.3) | 106 (0.3) |
| TCHAOUDJO | .. | .. | .. |  | .. | .. | .. |  | 919 (2.6) | 302 (0.8) | 161 (0.4) |  | 492 (1.4) | 97 (0.3) | 63 (0.2) | 38 (0.1) |  | 392 (1.1) | 69 (0.2) | 62 (0.2) |  | 278 (0.8) | 86 (0.2) | 67 (0.2) |  | 228 (0.6) | 39 (0.1) | 33 (0.1) | 47 (0.1) |
| Total | .. | .. | .. |  | .. | .. | .. |  | 2913 (2.6) | 705 (0.6) | 339 (0.3) |  | 1995 (1.7) | 360 (0.3) | 243 (0.2) | 190 (0.2) |  | 1957 (1.7) | 443 (0.4) | 361 (0.3) |  | 1299 (1.1) | 418 (0.3) | 400 (0.3) |  | 929 (0.7) | 256 (0.2) | 225 (0.2) | 228 (0.2) |
| *KARA* | NA | NA | NA |  | NA | NA | NA |  |  |  |  |  |  |  |  |  |  |  |  |  |  |  |  |  |  |  |  |  |  |
| ASSOLI | .. | .. | .. |  | .. | .. | .. |  | 250 (2.9) | 144 (1.6) | 16 (0.2) |  | 155 (1.6) | 40 (0.4) | 26 (0.3) | 18 (0.2) |  | 50 (0.5) | 32 (0.3) | 26 (0.3) |  | 71 (0.7) | 39 (0.4) | 114 (1) |  | 38 (0.3) | 19 (0.2) | 11 (0.1) | 16 (0.1) |
| BASSAR | .. | .. | .. |  | .. | .. | .. |  | 679 (3.1) | 110 (0.5) | 33 (0.1) |  | 470 (2.1) | 144 (0.6) | 156 (0.7) | 72 (0.3) |  | 288 (1.4) | 123 (0.6) | 140 (0.6) |  | 492 (2.2) | 220 (1) | 183 (0.8) |  | 389 (1.7) | 206 (0.9) | 214 (0.9) | 166 (0.7) |
| BINAH | .. | .. | .. |  | .. | .. | .. |  | 603 (4.7) | 126 (1) | 55 (0.4) |  | 587 (4.4) | 156 (1.2) | 231 (1.7) | 128 (0.9) |  | 468 (3.8) | 229 (1.8) | 63 (0.9) |  | 466 (3.5) | 202 (1.4) | 219 (1.6) |  | 227 (1.6) | 139 (1) | 62 (0.4) | 59 (0.5) |
| DANKPEN | .. | .. | .. |  | .. | .. | .. |  | 1646 (6) | 335 (1.1) | 113 (0.4) |  | 791 (2.6) | 353 (1.2) | 236 (0.8) | 92 (0.3) |  | 423 (1.5) | 209 (0.7) | 148 (0.5) |  | 419 (1.5) | 382 (1.3) | 356 (1.2) |  | 378 (1.3) | 294 (0.9) | 273 (0.8) | 200 (0.6) |
| DOUFELGOU | .. | .. | .. |  | .. | .. | .. |  | 151 (2.6) | 41 (0.3) | 21 (0.1) |  | 402 (2.7) | 63 (0.4) | 77 (0.5) | 26 (0.2) |  | 146 (1.1) | 41 (0.3) | 47 (0.3) |  | 186 (1.4) | 62 (0.4) | 81 (0.5) |  | 130 (0.9) | 53 (0.3) | 30 (0.2) | 33 (0.2) |
| KERAN | .. | .. | .. |  | .. | .. | .. |  | 612 (3.5) | 139 (0.7) | 60 (0.3) |  | 470 (2.2) | 110 (0.5) | 77 (0.4) | 42 (0.2) |  | 97 (0.6) | 32 (0.2) | 41 (0.2) |  | 231 (1.1) | 71 (0.3) | 46 (0.2) |  | 145 (0.7) | 49 (0.2) | 22 (0.1) | 34 (0.1) |
| KOZAH | .. | .. | .. |  | .. | .. | .. |  | 837 (2.2) | 257 (0.7) | 129 (0.3) |  | 698 (1.7) | 247 (0.6) | 168 (0.4) | 108 (0.3) |  | 466 (1.2) | 182 (0.4) | 198 (0.5) |  | 509 (1.2) | 280 (0.6) | 420 (1) |  | 373 (0.9) | 218 (0.5) | 169 (0.3) | 127 (0.3) |
| Total | .. | .. | .. |  | .. | .. | .. |  | 4778 (3.6) | 1152 (0.8) | 427 (0.3) |  | 3573 (2.3) | 1113 (0.7) | 971 (0.6) | 486 (0.3) |  | 1938 (1.4) | 848 (0.6) | 663 (0.4) |  | 2374 (1.6) | 1256 (0.8) | 1419 (0.9) |  | 1680 (1.1) | 978 (0.6) | 781 (0.4) | 635 (0.4) |
| *SAVANES* |  |  |  |  |  |  |  |  |  |  |  |  |  |  |  | NA |  |  |  |  |  |  |  | NA |  |  |  |  |  |
| CINKASSE | 321 (2.5) | 81 (0.6) | 11 (0.1) |  | 253 (1.7) | 93 (0.6) | 140 (0.9) |  | 295 (1.9) | 125 (0.8) | 50 (0.3) |  | 287 (2.1) | 164 (1) | 89 (0.5) | .. |  | 290 (1.8) | 148 (0.9) | 165 (1) |  | 220 (1.5) | 165 (1) | .. |  | 174 (1.1) | 157 (0.9) | 106 (0.6) | 87 (0.5) |
| KPENDJAL | 1103 (3.7) | 588 (1.9) | 43 (0.1) |  | 1174 (3.2) | 920 (2.4) | 575 (1.5) |  | 1125 (3.5) | 514 (1.5) | 173 (0.5) |  | 1176 (4) | 589 (1.7) | 290 (0.8) | .. |  | 976 (2.8) | 523 (1.4) | 378 (1) |  | 732 (2) | 492 (1.4) | .. |  | 875 (2.3) | 538 (1.4) | 281 (0.7) | 265 (0.6) |
| OTI |  |  |  |  | 1063 (2.7) | 124 (0.3) | 95 (0.2) |  | 375 (1.1) | 107 (0.3) | 53 (0.1) |  | 301 (1.4) | 37 (0.1) | 70 (0.2) | .. |  | 192 (0.5) | 54 (0.1) | 12 (0.1) |  | 75 (0.2) | 36 (0.1) | .. |  | 162 (0.4) | 105 (0.2) | 71 (0.1) | 38 (0.1) |
| TANDJOARE | 708 (3.7) | 515 (2.4) | 52 (0.7) |  | 576 (2.6) | 306 (1.3) | 149 (0.6) |  | 569 (2.7) | 181 (0.8) | 79 (0.4) |  | 191 (2.1) | 134 (0.6) | 92 (0.4) | .. |  | 252 (1.1) | 230 (1) | 155 (0.6) |  | 387 (1.6) | 94 (0.4) | .. |  | 235 (0.9) | 106 (0.4) | 44 (0.2) | 44 (0.2) |
| TONE | 871 (1.8) | 387 (0.7) | 152 (0.3) |  | 1190 (2.1) | 240 (0.4) | 281 (0.5) |  | 1212 (2.3) | 442 (0.8) | 163 (0.3) |  | 1005 (1.9) | 295 (0.5) | 201 (0.4) | .. |  | 565 (1) | 265 (0.5) | 239 (0.4) |  | 544 (0.9) | 375 (0.6) | .. |  | 489 (0.8) | 395 (0.6) | 119 (0.2) | 98 (0.1) |
| Total | 3003 (2.7) | 1571 (1.3) | 258 (0.2) |  | 4256 (2.5) | 1683 (1) | 1240 (0.7) |  | 3576 (2.3) | 1369 (0.9) | 518 (0.3) |  | 2960 (2.3) | 1219 (0.7) | 742 (0.4) | .. |  | 2275 (1.4) | 1220 (0.7) | 949 (0.6) |  | 1958 (1.1) | 1162 (0.6) | .. |  | 1935 (1) | 1301 (0.7) | 621 (0.3) | 532 (0.3) |
| Overall total | 3003 (2.7) | 1571 (1.3) | 258 (0.2) |  | 4256 (2.5) | 1683 (1) | 1240 (0.7) |  | 11267 (2.8) | 3226 (0.8) | 1284 (0.3) |  | 8528 (2.2) | 2692 (0.6) | 1956 (0.4) | 676 (0.2) |  | 6170 (1.5) | 2511 (0.6) | 1973 (0.5) |  | 5631 (1.3) | 2836 (0.6) | 1819 (0.6) |  | 4544 (1) | 2535 (0.5) | 1627 (0.3) | 1395 (0.3) |
